# Amyloid Dysmetabolism Relates to Reduced Glucose Uptake in White Matter Hyperintensities

**DOI:** 10.3389/fneur.2016.00209

**Published:** 2016-11-21

**Authors:** Lisa Flem Kalheim, Per Selnes, Atle Bjørnerud, Christopher Coello, Kjetil Vegge, Tormod Fladby

**Affiliations:** ^1^Department of Neurology, Akershus University Hospital, Lørenskog, Norway; ^2^Institute of Clinical Medicine, University of Oslo, Oslo, Norway; ^3^The Intervention Centre, Oslo University Hospital, Oslo, Norway; ^4^Preclinical PET/CT, Institute of Basic Medical Sciences, University of Oslo, Oslo, Norway; ^5^Department of Radiology, Akershus University Hospital, Lørenskog, Norway

**Keywords:** Alzheimer’s disease, cerebrovascular disease, white matter, PET, cerebrospinal fluid

## Abstract

Alzheimer’s disease (AD) is the most prevalent neurodegenerative disorder and cause of dementia and is characterized by amyloid plaques and neurofibrillary tangles. AD has traditionally been considered to primarily affect gray matter, but multiple lines of evidence also indicate white matter (WM) pathology and associated small-vessel cerebrovascular disease. WM glucose delivery and metabolism may have implications for local tissue integrity, and [^18^F]-fluorodeoxyglucose positron emission tomography (FDG-PET) may be helpful to assess neuroglial and axonal function in WM. Hypothesizing that affection of oligodendroglia will be associated with loss of glucose uptake, we aimed to investigate glucose metabolism in magnetic resonance imaging (MRI) white matter hyperintensities (WMHs) and normal-appearing WM in patients with and without evidence of amyloid plaques. Subjects with mild cognitive impairment or subjective cognitive decline were included and dichotomized according to pathological (Aβ+) or normal (Aβ−) concentrations of cerebrospinal fluid amyloid-β 1–42. A total of 50 subjects were included, of whom 30 subjects were classified as Aβ(+) and 20 subjects as Aβ(−). All subjects were assessed with MRI and FDG-PET. FDG-PET images were corrected for effects of partial voluming and normalized to cerebellar WM, before determining WMH FDG-uptake. Although there were no significant differences between the groups in terms of age, WMH volume, number of individual WMHs, or WMH distribution, we found significantly lower (*p* = 0.021) FDG-uptake in WMHs in Aβ(+) subjects (mean = 0.662, SD = 0.113) compared to Aβ(−) subjects (mean = 0.596, SD = 0.073). There were no significant group differences in the FDG-uptake in normal-appearing WM. Similar results were obtained without correction for effects of partial voluming. Our findings add to the evidence for a link between Aβ dysmetabolism and WM pathology in AD.

## Introduction

Although traditionally considered two distinct entities, accumulating evidence links Alzheimer’s disease (AD) and cerebrovascular disease (CVD) (1). White matter (WM) hyperintensities (WMHs) of presumed vascular origin (2) are commonly recognized as markers of small-vessel CVD and have been associated with mild cognitive impairment (MCI) and AD (3, 4). ApoEϵ4 alleles, a strong genetic determinant of AD risk, have been linked to both AD-related pathology (i.e., amyloid deposition) (5) and WMHs, particularly in extensive forms and combined with vascular risk factors (6, 7). However, the mechanisms linking the diseases remain unresolved (8).

Alzheimer’s disease is characterized by amyloid plaques and neurofibrillary tangles (9) formed by abnormal deposition of extracellular amyloid-β peptide (Aβ) and intraneuronal hyperphosphorylated tau, respectively. Also, vascular Aβ deposition (amyloid angiopathy) is present in the vast majority of subjects with AD (10). The concentration of Aβ in cerebrospinal fluid (CSF) decreases years before the appearance of clinical symptoms in AD (11), low levels being considered a key biomarker, and has high diagnostic accuracy for AD [reviewed in Blennow et al. (12)]. Low CSF Aβ shows inverse correlations with positive amyloid positron emission tomography (PET) scans (13) and amyloid plaques demonstrated at autopsy (14), probably reflecting cortical Aβ deposition.

According to the amyloid cascade hypothesis (15, 16), Aβ dysmetabolism initiates the AD pathologic cascade resulting in neuronal loss and dysfunction, i.e., neurodegeneration. Cerebral hypometabolism, a neurodegenerative marker, results in reduced uptake of [^18^F]-fluorodeoxyglucose (FDG)-PET. In AD, mostly gray matter glucose metabolism has been studied; however, WM abnormalities are also common (17). Although vascular disease and WMHs have been associated with frontal hypometabolism (18), to our knowledge, no studies have investigated FDG-uptake in WM and WMHs in AD.

Demyelination and axonal rarefaction characterize WMHs, and we recently demonstrated reduced WMH tissue integrity in the presence of amyloid dysmetabolism, as measured by diffusion tensor imaging (19). With limited glycolytic capacity, axonal compartments depend on mitochondrial activity (20, 21). WM neuroglia metabolize glucose and subserve axonal energy requirements probably by way of lactate transfer (22), long-term energy deprivation leading to axonal dysfunction, neuronal death, and loss of oligodendrocytes (23, 24). Thus, WM glucose supply and uptake depends on neuroglial function, and FDG-PET optimized for WM may be a helpful tool to assess WM function and integrity.

We hypothesized more pronounced hypometabolism within WMHs in amyloid-positive compared to amyloid-negative subjects (here, defined by the level of CSF Aβ_42_). For comparison, we further analyzed FDG-PET uptake in normal-appearing white matter (NAWM).

## Materials and Methods

### Subjects

**Figure 1:**
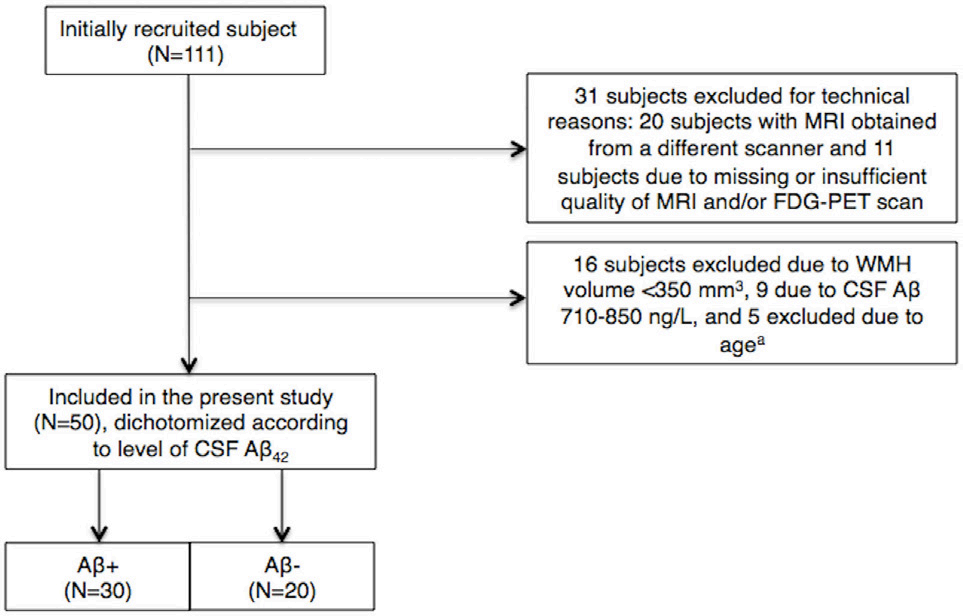
**Inclusions and exclusions in the cohort**. ^a^5 subjects of >73 or <44 years of age were excluded to limit between-group age differences. CSF, cerebrospinal fluid; Aβ, amyloid β-peptide; WMH, white matter hyperintensity.

Subjects referred to a university hospital-based neurological outpatient memory clinic between 2005 and 2013 were routinely evaluated for study inclusion (Figure 1). Written informed consent was obtained from all participants prior to enrollment. Inclusion criteria were age 40–79 years, subjective cognitive complaints ≥6 months, and absence of dementia.

Included subjects were classified as MCI or subjective cognitive decline (SCD) according to the Global Deterioration Scale (GDS) 2 or 3 (25, 26), as determined from clinical interview and screening tests: mini–mental state examination (MMSE) (27), the neurobehavioral cognitive status examination (Cognistat) (28), I-Flex (word fluency, interference, and numeral–letter items) (29, 30). SCD was defined as GDS 2, based on published cutoffs on the listed screening tests (≥28 for MMSE, <2 for I-Flex, above mild impairment on all the items of Cognistat). MCI was defined as GDS 3, based on scores below cutoff on one or more of the screening tests (30). Subjects classified as GDS 3/MCI fulfill general criteria for MCI, as revised by Petersen et al. (31). The final diagnoses were established in consensus conferences of physicians and neurologists at a university hospital. Participants with GDS >3 and clinical dementia rating >0.5 (32) were considered demented, i.e., with evidence of significant impairment in activities of daily living, and excluded from the study. Subjects with a history of learning disabilities, established psychiatric comorbidity, anoxic brain damage, drug abuse, or solvent exposure were excluded. All participants had a standardized clinical assessment including neurological examinations, magnetic resonance imaging (MRI), FDG-PET, and lumbar puncture.

Cerebrospinal fluid Aβ_42_ <710 ng/L was considered pathological, or Aβ(+), according to our research criteria. This CSF Aβ_42_ cutoff has recently been validated against Flutemetamol-PET (amyloid-PET) in an extension of the current cohort (Almdahl et al., 2016, submitted). Adding variance for measurement technique places the potential variance-dependent CSF Aβ_42_ cutoff above 710 ng/L. To avoid including false negatives (subjects with amyloid pathology) in the control [Aβ(−)] group, subjects with CSF Aβ_42_ levels between 710 and 850 ng/L were excluded. Subjects with CSF Aβ_42_ >850 ng/L were classified as Aβ(−) and used as a control group. For the present study, data were obtained from 50 subjects selected based on CSF Aβ_42_ <710 or >850 ng/L and WMH volume >350 mm^3^, of whom 29 were females and 21 males, 30 Aβ(+), and 20 Aβ(−). Subjects with WMH volume <350 mm^3^ were excluded as we considered this to be the minimum volume required for valid analyses. Between-group age difference was limited by excluding subjects aged >73 and <44 years.

Based on the Norwegian Health and Research Act and the Helsinki Declaration, the study was approved by Regional Committee for Medical and Health Research Ethics, South East Norway.

### MRI/FDG-PET Acquisition

Magnetic resonance imaging was performed using a Siemens Espree 1.5-T scanner. A 3D magnetization-prepared rapid gradient echo (MP-RAGE) T1-weighted sequence was obtained (TR/TE/inversion time/flip angle = 2400/3.65/1000/8°, matrix = 240 × 192, 160 sagittal slices, thickness = 1.2 mm, in-plane resolution of 1 mm × 1.2 mm). A 2D axial fluid-attenuated inversion recovery (FLAIR) image with the following parameters was obtained: TR/TE/TI = 13,420/121/2500, 36 slices, spaced at 3.0 and 3.9 mm slice thickness.

Fluorodeoxyglucose-PET/CT imaging was performed with a Siemens Biograph 16 PET/CT scanner. All subjects fasted at least 4 h before image acquisition and received a bolus injection of 218 MBq (±23 MBq) FDG tracer followed by 45 min of rest before being positioned head-first supine in the scanner. Prior to the PET acquisition, a low-dose CT scan for attenuation correction was acquired. Patients were scanned for 15 min in 3D mode using one bed position (axial range = 16 cm). Data were corrected for random events, dead time, attenuation (CT-derived μ-map), scatter (model based), and decay. PET volumes were reconstructed using an iterative algorithm (OSEM 2D, four iterations, eight subsets), and a post-reconstruction 3D Gaussian filter 3.5 mm full-width half maximum was applied. The axial image format was 256 × 256 (pixel size: 2.67 mm × 2.67 mm), with a slice thickness of 2.00 mm. Blood glucose at FDG injection time was measured routinely, and the subject was excluded if blood glucose concentration exceeded 8.0 mmol/L.

### Image Processing

Measurement accuracy of PET imaging tracer uptake may be limited by a relatively poor scanner resolution and by reconstruction algorithm, resulting in partial voluming effects (i.e., spillover effect from high to adjacent low [^18^F] FDG-uptake regions). To adjust for this, and to obtain correct quantification of glucose metabolism (i.e., FDG-PET uptake), we recently developed a partial voluming correction method, applied herein (33). Whole head PET volumes were co-registered to the anatomical volume using a 6-parameter rigid body spatial registration as implemented in the Spatial Parametrical Mapping (SPM 8, Wellcome Trust Centre for Neuroimaging, UCL, UK) co-registration tool. PET volumes were resliced into 1 mm MRI space using a spline interpolation. For each subject, a voxel-based intensity normalization to the mean uptake in the cerebellar WM was performed. (Prior to intensity normalization, the cerebellar WM mask was eroded to avoid effects of partial voluming, inaccurate segmentation, or co-registration.) Intensity normalized volumes were used for all further analyses.

**Figure 2:**
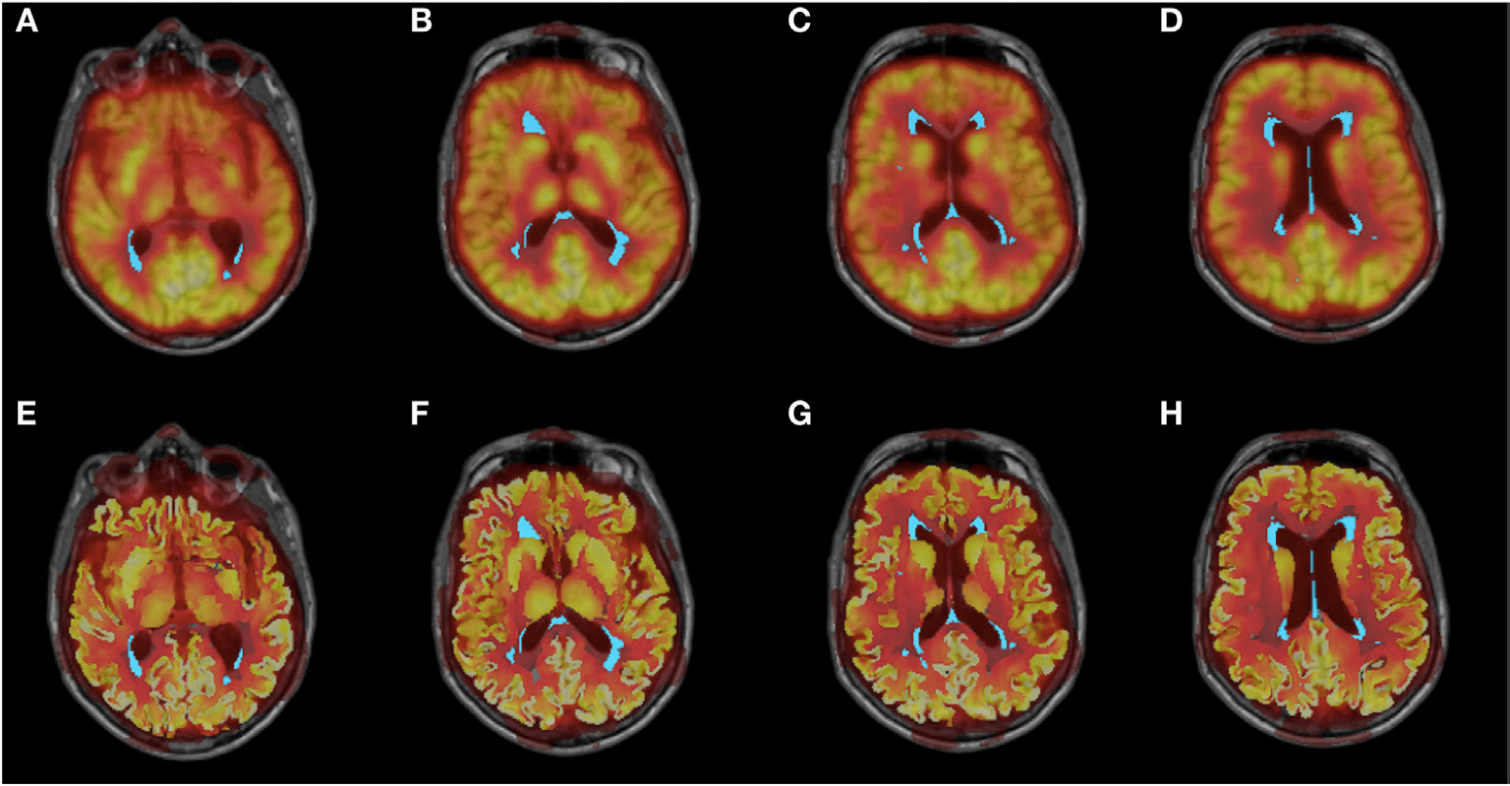
**Co-registered structural MRI, WMH segmentation (cyan), and partial voluming uncorrected (A–D) and corrected (E–H) FDG-PET images**.

Further, the FDG-PET and structural images were co-registered to high-resolution 3D T1-weighted MRI scans for WMH segmentation and measurements (Figure 2). FDG-PET analyses and calculations were performed using The Oxford Centre for Functional MRI of the Brain (FMRIB) Software Library (FSL) version 5.0 (34, 35).

A recently developed in-house object-based supervised machine-learning algorithm was used for automated segmentation and quantification of WMHs based on FLAIR image intensity and masks of tissue types (36). All segmentations were manually inspected for accuracy and edited when necessary. For each subject, the number of non-interconnected hyperintense areas was further determined, as well as periventricular WMH volume (≤10 mm from the ventricles). The reconstruction and segmentation of WM was performed with the FreeSurfer image analysis suite version 5.3.0 (http://surfer.nmr.mgh.harvard.edu/). NAWM was defined by subtracting WMH from the total WM. FDG-PET measurements in NAWM were extracted using FSL.

### Cerebrospinal Fluid Analysis

Lumbar puncture was performed consecutively after inclusion, in the L3/L4 or L4/L5 interspace at a standardized time of day (around noon). CSF samples were examined for levels of Aβ_42_, total microtubule-associated protein Tau (T-tau), and Tau phosphorylated at threonine 181 (P-tau) with commercially available enzyme-linked immunosorbent assay kits (Innogenetics, Belgium, presently Fujirebio Europe).

The procedure was carried out in accord with the manufacturers’ procedures at the national reference laboratory for these tests at the Department of Interdisciplinary Laboratory Medicine and Medical Biochemistry, Akershus University Hospital. Although measurements of CSF AD biomarkers show between-laboratory and batch-to-batch assay variability (37), the national reference laboratory is part of the European multicenter project “Biomarkers for Alzheimer’s disease and Parkinson’s disease” (BIOMARKAPD), funded by EU Joint Programme-Neurodegenerative Disease Research (JPND), for standardization of analytical methods and laboratory procedures to increase its accuracy with regard to CSF biomarkers of neurodegenerative diseases.

### Statistical Analysis

Group differences in demographic and clinical features were assessed using χ^2^ tests for categorical data and independent-samples *T*-test or Mann–Whitney *U* test for continuous data. The WMH volume distribution was skewed and therefore logarithmically transformed prior to this analysis.

#### FDG-PET Uptake Differences in WMHs

The partial voluming corrected FDG-PET signal in WMHs was log-transformed prior to regression to meet the assumptions of linear regression and then adjusted for age and WMH volume. The ensuing residuals were normally distributed (as assessed by the Kolmogorov–Smirnov test and visual inspection of the histograms) and therefore used for further statistical analyses. Conducting independent-samples *T*-test, we compared group differences (i.e., Aβ+ vs. Aβ−) in PET signal in WMHs with a significance level set at *p* < 0.05.

#### Associations between Potential Confounders and FDG-PET Uptake in WMHs

Linear regression analyses were carried out for both groups (i.e., Aβ+ and Aβ−). Using linear regression, we analyzed potential effects of the presence of ApoEϵ4 alleles. Although included as covariates in our main analyses, we further analyzed the effects of age and WMH volume on the FDG-PET signal in WMHs by means of linear regression with the FDG-PET signal as the dependent variable, and age and WMH volume as independent variables. In a separate linear regression model, sex was also included to assess potential associations with the FDG-uptake in WMHs.

#### WMH Location and Size

To assess whether our results could be influenced by group differences in WMH location (periventricular vs. subcortical) or individual WMH size, we determined the mean size of the per-subject of non-interconnected WMHs and the periventricular WMH volume. The volume measures were log-transformed prior to analyses. Individual mean WMH sizes were adjusted for age and total WMH volume, and the resulting residuals were normally distributed (assessed by the Kolmogorov–Smirnov test and inspection of histograms). We were unable to adjust periventricular WMH volume for both age and total WMH volume because this analysis did not fulfill the assumptions of regression, i.e., the resulting residuals were not normally distributed. We therefore adjusted periventricular WMH volume for age (with resultant normally distributed residuals). Group differences were assessed with independent-samples *T*-test with a significance level of *p* < 0.05.

#### FDG-PET Uptake in NAWM

In comparison to effects in WMHs, we analyzed the FDG-PET signal in NAWM. The FDG-PET signal was adjusted for age and WMH volume by a linear regression model, and the resulting residuals were normally distributed as assessed by Kolmogorov–Smirnov test and visual inspection of histograms. Conducting an independent-samples *T*-test, we compared FDG-PET signal in NAWM between the Aβ(+) and Aβ(−) group. Significance was determined by a *p*-value < 0.05.

All statistical analyses were performed with SPSS version 22 (SPSS Inc., Chicago, IL, USA).

## Results

Demographic and clinical data are shown in Table 1. Analyses revealed neither statistically significant group differences in terms of sex, age, or WMH volume nor were there significant group differences in terms of diagnoses (SCD/MCI).

**Table 1:** **Demographic and clinical data**.

	Aβ(+) (*n* = 30)	Aβ(−) (*n* = 20)	*p*-Value (Aβ+ vs. Aβ−)
Age, years	63.77 (6.99)	59.85 (7.29)	0.062^a^
Women/men, *n*	19/11	10/10	0.349^b^
MMSE	27.70 (1.34)	28.15 (1.50)	0.254^c^
SCD/MCI diagnoses, *n* (%)	3 (10.0)/27 (90.0)	4 (20.0)/16 (80.0)	0.281^b^
CSF Aβ_42_, ng/L	521.07 (133.23)	1036.80 (120.82)	<0.001^a^^,^*
WMH volume, mm^3^	5542.76 (7321.89)	3345.38 (5063.31)	0.291^a^^,^^d^

*Values are given in mean (SD) unless otherwise indicated*.

*^a^Calculated using independent-samples T-test*.

*^b^Calculated using Pearson chi-square test*.

*^c^Calculated using Mann–Whitney U test*.

*^d^p-Value for log transformed WMH volume adjusted for age*.

*Significant difference between the two groups.

*CSF, cerebrospinal fluid; Aβ, amyloid β-peptide; WMH, white matter hyperintensity; MMSE, mini-mental state examination; MCI, mild cognitive impairment; SCD, subjective cognitive decline*.

### FDG-PET Uptake Differences in WMHs

**Figure 3:**
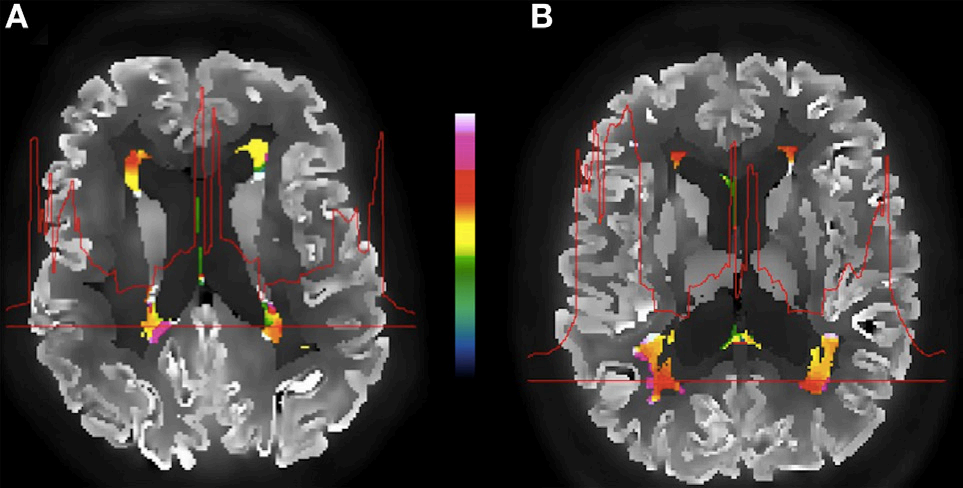
**Example of an amyloid-positive (A) vs. amyloid-negative (B) subject and the corrected FDG-PET images, WMH segmentation (color), and the metabolic profile (red line) for the selected WMH section across the brain**. The color bar indicates normalized relative uptake.

**Figure 4:**
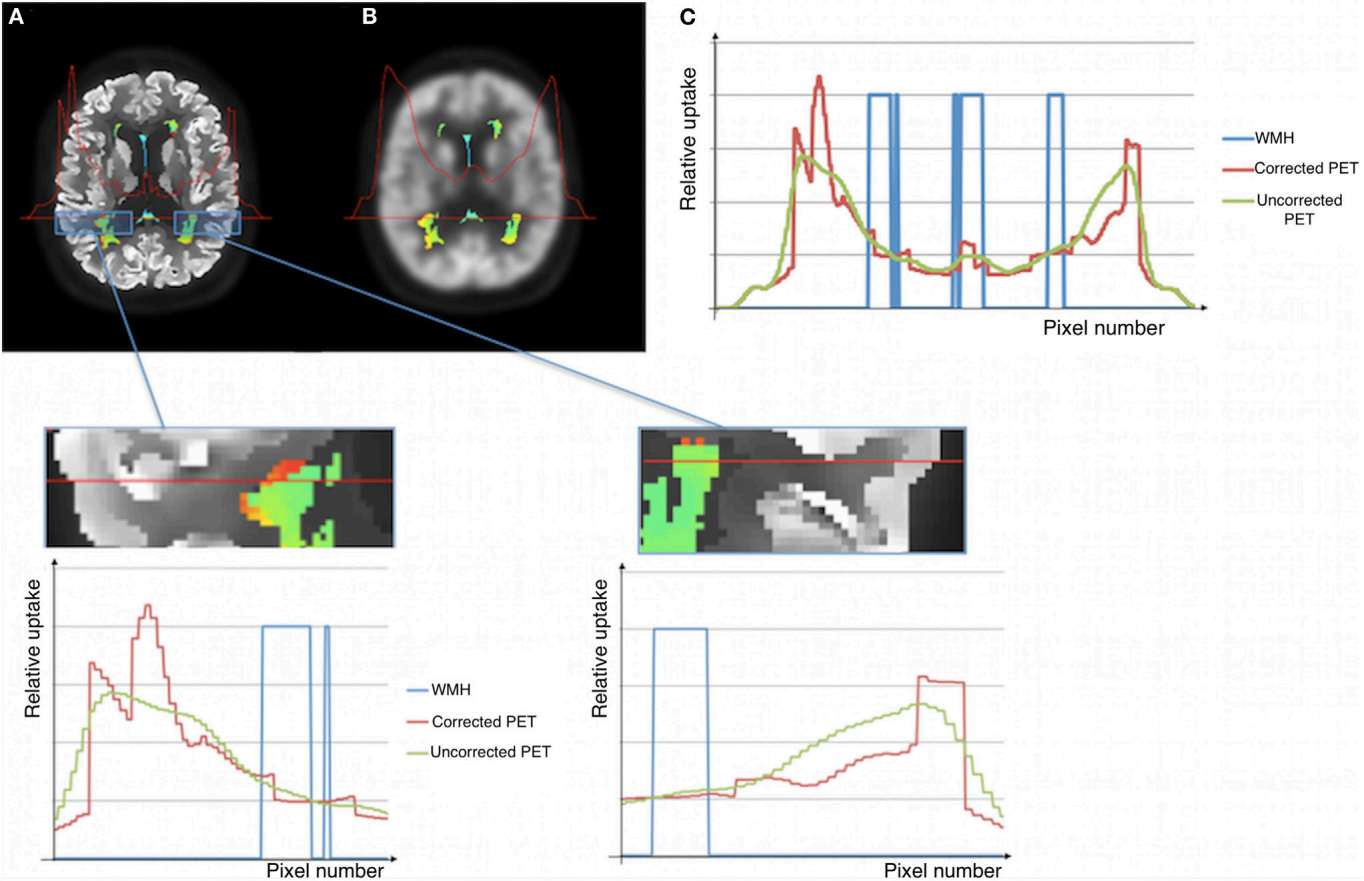
**Illustration of corrected (A) and uncorrected (B) images for effects of partial voluming**. WMHs PET intensities and the metabolic profile for the selected WMH section across the brain shown in **(C)**.

To investigate the effects of amyloid pathology on WMH glucose uptake, we analyzed the FDG-PET signal in WMHs in Aβ(+) and Aβ(−) subjects (Figure 3). Including potential confounders such as age and WMH volume, analyses of normalized, partial voluming corrected FDG-PET signal in WMHs revealed significantly reduced activity (*p* = 0.021) in Aβ(+) (mean = 0.596; SD = 0.073) relative to Aβ(−) (mean = 0.662, SD = 0.113). Cohen’s effect size value (*d* = −0.67) indicates moderate to high effect. Inclusion of ApoEϵ4-status as an additional covariate did not alter the findings. Our findings were also consistent in additional analyses without adjustment for age and WMH volume. When analyzing the uncorrected FDG-PET signal (i.e., without correcting for effects of partial voluming), adjusted for age and WMH volume, the significant results were consistent, i.e., significant difference (*p* = 0.014) when comparing Aβ(+) (mean = 0.598, SD = 0.074) and Aβ(−) subjects (mean = 0.676, SD = 0.113). Although consistent between-group differences were observed using uncorrected FDG-PET images, the intensity profiles differ slightly between corrected and uncorrected PET images (Figure 4).

### Associations between Potential Confounders and FDG-PET Uptake in WMHs

We investigated potential bias by performing linear regression analyses with different potential confounders. Results are summarized in Table 2. Linear regression analyses revealed no significant effect of sex, age, or WMH volume on the FDG-PET signal in WMHs in neither of the groups. Therefore, we did not correct for sex in our analyses. Using age and WMH volume as covariates, linear regression analyses further revealed no significant associations between ApoEϵ4-status and WMHs FDG-PET uptake in Aβ(+) or Aβ(−).

**Table 2:** **Effects of age, WMH volume, and ApoE on the FDG-PET uptake in WMHs**.

Independent variables	Dependent variables					
	FDG-PET uptake in WMHs					
	Aβ(+)			Aβ(−)		
	Beta	*p*	*R*^2^	Beta	*p*	*R*^2^
WMH volume	0.187	0.369	0.030	−0.278	0.259	0.075
Age	−0.071	0.732		0.069	0.776	
WMH volume	0.133	0.533		−0.269	0.301	
Age	−0.054	0.795	0.067	0.064	0.800	0.076
Sex	0.198	0.322		0.034	0.895	
WMH volume	0.138	0.500		−0.353	0.177	
Age	−0.041	0.842	0.063	0.072	0.767	0.126
ApoE4	0.20	0.300		−0.24	0.351	

*Associations between age, WMH volume, and ApoE, and the WMH FDG-PET uptake were determined by means of linear regression*.

*FDG-PET, [^18^F]-fluorodeoxyglucose positron emission tomography; WMH, white matter hyperintensity; Aβ, amyloid β-peptide; ApoE4, apolipoprotein E4*.

### WMH Location and Size

We compared the WMH location and individual WMH size, as these factors could affect the primary analysis. With age and total WMH volume as covariates, we found no significant differences when comparing the mean per-subject size of individual WMHs (*p* = 0.545) of the Aβ(+) (mean = 101 mm^3^, SD = 67 mm^3^) and Aβ(−) (mean = 75 mm^3^, SD = 28 mm^3^) groups. In addition, when corrected for age, analyses of the periventricular WMH volume did not reveal significant differences (*p* = 0.117) between Aβ(+) (mean = 4942 mm^3^, SD = 6611 mm^3^) and Aβ(−) (mean = 2639 mm^3^, SD = 3532 mm^3^). These results were consistent also without adjustment for covariates.

### FDG-PET Uptake in NAWM

The normalized FDG-PET signal in NAWM in both groups was further analyzed in comparison to the uptake in WMHs. Using age and WMH volume as covariates, we found no significant differences (*p* = 0.296) in NAWM when comparing Aβ(+) (mean = 0.910, SD = 0.059) and Aβ(−) (mean = 0.929, SD = 0.066). Although the FDG-PET signal was lower in the Aβ(+) group, the results remained non-significant also without adjusting for age and WMH volume.

## Discussion

To our knowledge, we are the first to assess the FDG-PET signal within WMHs and also the association with amyloid pathology. The present results support an association between amyloid dysmetabolism and WMHs, as the lowest WMH metabolism is seen in cases with amyloid pathology. This was not encountered in NAWM and may therefore be construed as specific for WMHs.

Cortical hypometabolism associated with WMHs differs from the temporoparietal pattern typically seen in AD (18, 38). It has been suggested that the association between WMHs and cerebral hypometabolism reflects synaptic hypoactivity (i.e., diaschisis) as a result of CVD, rather than being related to amyloid pathology (39). However, in the present study, Aβ(+) subjects had significantly lower FDG-PET signal within WMHs compared to Aβ(−) subjects, suggesting a link between amyloid dysmetabolism and glucose utilization and metabolism. While ApoEϵ4 alleles are associated with AD and WMHs, this relationship appears to be more prominent in late-stage cases, whereas the present results suggest that amyloid is more related to initial WMH severity in terms of affected metabolism (7, 40).

The pathogenic interactions between CVD and AD are complex, and the exact mechanisms remain unclear. It has been hypothesized that vascular risk factors and resulting vascular injury, lead to cerebral hypoperfusion and impair the blood–brain barrier function (41). Consequently, acceleration of Aβ production and impairment of its clearance may occur, leading to Aβ accumulation. However, associations between CVD and AD are observed also in populations without substantial vascular comorbidities (42), supporting an underlying heterogeneity of WMHs in AD, i.e., not only related to vascular factors and small-vessel CVD.

Conversely, Aβ may be involved in the vascular alterations observed in AD, as hypothesized herein. Cerebral amyloid angiopathy (CAA), characterized by amyloid deposits in leptomeningeal and cortical arteries and arterioles are associated with WMHs (43). CAA and ischemic brain injury coexist (44), and studies have shown that WMHs in AD (3), as well as in CAA (45, 46), have a predominant posterior cerebral distribution. Therefore, it is possible that vessel wall amyloid deposits may compromise perfusion and WMH metabolism in cases with low CSF Aβ.

Microscopically, the cerebral vasculature in AD presents with several pathological changes: degeneration of endothelial cells and surrounding pericytes, thickening of the capillary basement membrane, and, as the disease progresses, the cerebral blood flow (CBF) decreases (47). As glucose delivery and utilization are tightly coupled to regional CBF (48), CBF reduction may result in concurrently reduced glucose supply. The findings of more pronounced hypometabolism in the presence of amyloid pathology may therefore potentially result from amyloid-related hypoperfusion, and consequently hypometabolism, within WMHs.

The current findings are in line with our early work establishing associations between low CSF Aβ_42_ and WMHs, and also with our recently published data using MRI diffusion parameters to study WMH tissue integrity (19, 49). The latter data imply impaired microstructural integrity in WMHs in the presence of amyloid dysmetabolism, suggesting more pronounced oligodendroglial and axonal damage in the presence of amyloid pathology (19). Reduced glucose uptake in Aβ(+) subjects illustrate that WMH glucose metabolism is similarly affected, relating to impaired function of the WM components including neuroglia (22).

Patient cases at the SCD and MCI stages are etiologically heterogenous, also differing in FDG-uptake (50). However, subject selection to the current study is based on the CSF Aβ_42_ level and not on clinical diagnoses. Although the cortical FDG-PET signal in general may be lower in MCI, the proportion of SCD and MCI cases in the two study groups was similar, reducing a potential impact on our results. Therefore, and also to avoid overadjustment, patient stage was not included as a covariate in the current study.

Different spatial distribution of WMHs could be a potential bias. However, this was investigated in the same study population in a previous work (19), and no statistically significant group differences were observed in either of the cerebral lobes (i.e., frontal, parietal, temporal, occipital lobes) or cingulate. Although the phenomenon of WMH penumbra was recently described (51), as microstructural alterations were observed also in the vicinity of areas of definite WMHs, these areas was not included in our analyses but may be of interest for future studies.

The WMHs FDG-PET intensities are slightly different when comparing partial voluming corrected and uncorrected intensities (Figure 3), although the between-group differences are consistent. While the uncorrected images show a gradual metabolic reduction from the cortex toward the deeper brain regions, the corrected intensity profile demonstrates a clear-cut reduction corresponding to tissue borders (as the gray/WM border), probably giving a closer approximation to the actual cerebral metabolism. This shows that applying partial voluming correction methods, as herein, enables the use of optimized PET images (i.e., partial voluming corrected images) also for WM and WMHs analyses.

Although correction for effects of partial voluming was carried out, limitations relating to FDG-PET must also be considered when interpreting the results. Spillover from the ventricles could occur due to the large contrast between the WM and the ventricles; however, no statistically significant group differences in periventricular WMH volume were observed. In addition, in both groups there was a strong positive correlation (*r* > 0.960, *p* < 0.001) between periventricular WMH volume and the total WMH volume (a covariate in our analyses), in effect also controlling for this. Furthermore, while smaller individual WMHs may be more vulnerable to spillover from the presumptively higher FDG-uptake NAWM, we found no significant group differences in the mean size of the individual non-interconnected WMH size. As such, we consider the bias relating to these factors to be reduced.

Age, strongly related to WMHs, and WMH volume may impact on the present results, and these factors were included as covariates to remove potential bias. However, their effect on the FDG-PET signal in WMHs was non-significant, which is in line with our observations with diffusion tensor imaging (19). Hence, it is less likely our results are biased by these factors and the FDG-PET signal in WMHs may rather be influenced by amyloid-related pathology, as suggested here.

Both our earlier findings of Aβ pathology-associated reduced WMH structural integrity and the present findings of reduced WMH glucose metabolism implicate neuroglia and oligodendrocyte pathology, which could be induced by Aβ-related capillary pathology as described above. However, cortical and intraneuronal Aβ pathology could also induce neuronal and mitochondrial pathology (52), a downstream effect on neuroglia putatively making WM elements susceptible to cerebrovascular pathology in AD cases. Neuropathological and *in vivo* perfusion and amyloid imaging studies could serve to distinguish these hypotheses.

The limitations mentioned, together with a relatively small sample size in the current study, necessitate that findings are replicated in larger cohorts, and future studies are also warranted to further establish mechanisms linking Aβ pathology and WMHs. However, the current findings add to the pool of evidence linking CVD and AD pathomechanistically and may reflect the heterogeneity of the WMH etiopathology in AD, involving both Aβ pathology and small-vessel CVD.

## Author Contributions

LK: study concept and design, analysis and interpretation of the data, and further drafted the manuscript. AB: study concept and design, acquisition and interpretation of data (notably MRI data), and critical revision of the manuscript. CC: analysis and interpretation of data (notably PET data) and critical revision of the manuscript. TF: study concept and design, acquisition and interpretation of data, and critical revision of the manuscript. KV: acquisition and interpretation of data (notably MRI data) and revision of the manuscript. PS: study concept and design, acquisition, analysis, and interpretation of the data, and critical revision of the manuscript.

## Conflict of Interest Statement

The authors declare that the research was conducted in the absence of any commercial or financial relationships that could be construed as a potential conflict of interest.
